# Combined Pulmonary Fibrosis and Emphysema (CPFE): A “New” Smoking-Related Interstitial Lung Disease (ILD)

**DOI:** 10.3390/biomedicines13112703

**Published:** 2025-11-03

**Authors:** Carina Adina Afloarei, Tudor Birladeanu, Adriana Loredana Pintilie, David Toma, Dragos Traian Marius Marcu, Andreea Zabara Antal, Mihai Zabara, Radu Crisan Dabija

**Affiliations:** 1 Clinical Hospital of Pulmonary Diseases, 700116 Iasi, Romania; carina_adina98@yahoo.com (C.A.A.); birladeanu.tudor@yahoo.com (T.B.); pintilie.adriana.loredana.mg6.7@gmail.com (A.L.P.); tomadavidro@gmail.com (D.T.); andreeazabara@yahoo.com (A.Z.A.); radu.dabija@umfiasi.ro (R.C.D.); 2 Grigore T. Popa University of Medicine and Pharmacy, 700116 Iasi, Romania; mihai-lucian.zabara@umfiasi.ro; 3 Department of General Surgery and Liver Transplantation, St. Spiridon University Hospital, 700115 Iasi, Romania

**Keywords:** combined pulmonary fibrosis and emphysema (CPFE), interstitial lung disease (ILD), emphysema, idiopathic pulmonary fibrosis (IPF), high-resolution computed tomography (HRCT), pulmonary hypertension, risk factors, pathophysiology, prognosis, smoking

## Abstract

**Background:** Combined Pulmonary Fibrosis and Emphysema (CPFE) is a distinct syndrome characterized by upper-lobe emphysema and lower-lobe fibrosis, predominantly in older male smokers. Despite often preserved spirometric volumes, patients exhibit severely reduced diffusing capacity and high susceptibility to complications, including pulmonary hypertension (PH), acute exacerbations, and lung cancer, contributing to poor prognosis. **Purpose:** This review aims to synthesize current evidence on CPFE, focusing on clinical phenotype, functional impairment, differential diagnosis, complications, and emerging management strategies, highlighting distinctions from idiopathic pulmonary fibrosis (IPF) and chronic obstructive pulmonary disease (COPD). **Methods:** A narrative review of observational cohorts, retrospective series, and clinical studies examining CPFE patients was performed. Data on demographics, smoking history, symptomatology, pulmonary function, radiology, comorbidities, complications, and treatment approaches were extracted and integrated. **Results:** CPFE affects mainly males aged 65–70, with >90% reporting > 40 pack–years smoking history. Dyspnea is the cardinal symptom (>95%), often disproportionate to preserved FVC and TLC, accompanied by chronic cough in 30–70%. Exercise-induced desaturation is frequent, correlating with PH, observed in 47–90% of patients. Pulmonary function tests reveal preserved volumes, normal or near-normal FEV1/FVC, and severely reduced DLCO (35–45%), distinguishing CPFE from COPD and IPF. HRCT confirms the combined emphysematous and fibrotic pattern, critical for differential diagnosis. Acute exacerbations occur in 20–28% of cases, lung cancer in 22–46% (mostly squamous cell), and long-term oxygen therapy is required in >70%. Five-year survival is 35–55%, lower than emphysema alone and comparable or worse than IPF. Management focuses on smoking cessation, antifibrotics, oxygen therapy, and complication-specific treatments, and selected patients may undergo lung transplantation. **Conclusions:** CPFE is a clinically and functionally unique entity with a high burden of pulmonary and systemic complications. Accurate recognition using HRCT and DLCO, along with early intervention and tailored management, is essential to improve patient outcomes and guide prognostic stratification.

## 1. Introduction

Combined Pulmonary Fibrosis and Emphysema (CPFE) is a distinct clinical entity, first described by Cottin et al. in 2005, characterized by the association of emphysema (predominantly centrilobular/paraseptal, located in the upper lobes) and pulmonary fibrosis (often with a usual interstitial pneumonia pattern—UIP—predominantly in the lower lobes) in patients with a history of significant smoking [1,2,3].

From a functional perspective, there is a dissociation between spirometry results, which may be relatively preserved, and a disproportionately severe decrease in carbon monoxide diffusing capacity (DLCO) [1,3]. While emphysema causes hyperinflation and an increase in total lung volume, the associated fibrosis cancels this effect by restriction, resulting in near-normal spirometry values. However, the reduction in DLCO remains marked, reflecting both the loss of alveolar surface area through emphysema and the thickening of the alveolar–capillary barrier through fibrosis [1,4,5].

Although CPFE was initially described in the context of idiopathic pulmonary fibrosis (IPF), the syndrome has subsequently been identified in other interstitial lung diseases (ILDs), including chronic hypersensitivity pneumonitis, non-IPF interstitial pneumonias, ILDs associated with connective tissue diseases, and familial cases linked to genetic mutations (e.g., SFTPC) [2,6,7,8]. Genetic studies also suggest a role for telomere shortening in susceptibility to IPF and emphysema, an association that may contribute to the development of CPFE [9,10]. The main risk factor remains exposure to cigarette smoke, common to both emphysema and fibrosis [11,12,13]. Thus, CPFE should not be confused with COPD or isolated pulmonary fibrosis, which is a distinct entity with different prognostic implications and an increased risk of complications [2,3].

The cardinal symptom reported by patients is progressive dyspnea on exertion, frequently present from the early stages of the disease [1,14,15]. Given the nonspecific nature of dyspnea, common to both pulmonary and cardiac pathology, an extensive diagnostic protocol is required, including history, clinical examination, spirometry, DLCO, chest radiography, 6-min walk test, ECG, and, subsequently, high-resolution computed tomography (HRCT) [2,14,15]. However, early recognition of patients is often delayed, mainly due to the lack of universally accepted diagnostic criteria [2,15]. The clinical course is usually complicated by the development of pulmonary hypertension (PH) and lung cancer [3,4,16].

The mortality of patients with CPFE is substantially increased compared to that of patients with isolated IPF or COPD, mainly determined by the progression of respiratory failure, the development of PH, and the significantly increased risk of lung cancer [4,5,14,16].

## 2. Materials and Methods

A structured narrative review of the literature was conducted, based on the main international medical databases: PubMed, Scopus, and Web of Science. The search included publications from 2010 to 2025, using the following keywords and combinations of terms: “combined pulmonary fibrosis and emphysema”, “CPFE”, “interstitial lung disease”, “smoking-related fibrosis”, “pulmonary hypertension”, and “antifibrotic therapy”.

Inclusion criteria targeted completed clinical and observational studies with methodological validity and clear, up-to-date documentation of CPFE. Studies with small sample sizes or ongoing research were excluded to ensure the consistency and statistical relevance of the analyzed data.

No formal meta-analysis was performed; the information obtained from the studies was integrated into a narrative review aimed at highlighting concordant findings, discrepancies between studies, and underexplored aspects in the current literature. Statistical data reported in the original studies, such as prevalence estimates, pulmonary function parameters, and biomarker distributions, were extracted, combining quantitative findings with contextual interpretation to provide a coherent synthesis of current knowledge on CPFE.

## 3. Results

### 3.1. Historical Background

In 1990, Wiggins et al. evaluated a group of 8 smokers with severe dyspnea, in whom HRCT imaging revealed both upper lobe emphysema and lower lobe fibrosis [1]. Initially, these findings were treated as coincidental. In 2005, Cottin et al. established the definition of CPFE based on a retrospective study of 61 patients with upper lobe emphysema and diffuse lower lobe fibrosis on HRCT [2]. This study was the first attempt to define CPFE as a distinct clinical entity and emphasized the importance of recognizing the coexistence of emphysema and fibrosis, as well as the need to differentiate it from COPD and IPF. Given the two pathologies underlying the definition of CPFE, a retrospective study was conducted in 2011 comparing the clinical and pathophysiological characteristics of patients with CPFE and those with IPF and COPD. The results highlighted major differences in the pathophysiological processes and the need to recognize CPFE as a distinct clinical entity, due to the increased risk of unfavorable evolution [3]. The study advocated for recognizing the syndrome as a distinct entity, with a more reserved prognosis compared to isolated IPF or COPD. Subsequently, diagnostic and management guidelines were developed (ATS/ERS/JRS/ALAT, 2022), which emphasize the role of HRCT and pulmonary function tests for early patient identification and prevention of possible complications [2].

### 3.2. Epidemiology

Emphysema is common among smokers with interstitial lung disease. Estimates of prevalence depend on both the population studied and the definitions used and range from 8–67% among those with IPF [12]. There are geographical variations, with reported prevalences of 10–40% in Asia, 8–20% in Europe, and 3–8% in the United States [1,3,4,5].

The literature data is limited, given that we are addressing a pathology that is not sufficiently recognized in the medical world. Moreover, in the absence of a clear definition, various diagnostic criteria have been used, which prevents the determination of an accurate prevalence and incidence. Imaging studies conducted in cohorts of COPD patients suggest that between 4% and 9% of them have interstitial lung disease detected by HRCT, and 3–4% have advanced fibrotic changes [12,14]. Smokers can frequently develop a form of interstitial fibrosis associated with emphysema, without a pre-existing diagnosis. CPFE has also been reported in different types of ILDs: 28% of patients with idiopathic pulmonary fibrosis also have COPD, while in ILDs associated with autoimmune diseases, such as rheumatoid arthritis or scleroderma, up to 30% of patients present the CPFE phenotype, most of whom have a history of smoking and emphysema [16]. This variability highlights the difficulty of accurately estimating prevalence and the need for more rigorous recognition of CPFE in clinical practice.

Reference studies in the literature universally support that the syndrome occurs among older smokers, but variability exists between cohorts due to differences in syndrome definition, inclusion criteria, and diagnostic methods used. The lack of universally accepted criteria makes comparison of results and determination of true incidence difficult, representing a limitation in current epidemiological data.

### 3.3. Risk Factors

Patients with CPFE are male, frequently diagnosed between the 6th and 8th decades of life, with a history of exposure to cigarette smoke. Most patients have a history of between 40–60 pack–years, an exposure that facilitates the concomitant development of fibrosis and pulmonary emphysema [1]. Smoking is the main risk factor, through its effects on both the development of emphysema and the progression of fibrosis. There are exceptions, such as patients with connective tissue disease or hypersensitivity pneumonitis, who have a lower exposure to cigarette smoke [7].

Men are more likely to develop this condition, with this correlation being based on the more frequent history of smoking among the male population. Exposure to pollutants or a toxic occupational environment may also contribute to the development of CPFE through chronic inflammation, oxidative stress, and lung remodeling, although smoking remains the main risk factor [11,12].

Genetic mutations associated with the risk of developing this disease have been identified in genes such as MUC5B and TOLLIP [2]. These mutations do not trigger CPFE alone, but they increase the risk in the presence of factors such as smoking or occupational exposure. The presence of CPFE has been reported in carriers of mutations in surfactant-related genes [2,8] or those related to telomeres [9,10]. The phenomenon of telomere shortening is associated with both COPD and idiopathic pulmonary fibrosis and, consequently, there is a possibility that it may also be associated with CPFE [9,10].

CPFE remains a multifactorial disease, being in fact the result of an accumulation of genetic, environmental and behavioral factors. In clinical cohorts, over 90% of patients had significant exposure to cigarette smoke, and the prevalence in men is over 70% [1,2,12]. Recognition of these risk factors may influence the timing of diagnosis and may contribute to more careful monitoring of patients at high risk.

CPFE is associated with higher mortality compared with COPD or isolated pulmonary fibrosis, particularly through the presence of secondary PH, progressive respiratory failure, and an increased risk of lung cancer [2,3,4].

### 3.4. Pathophysiology

In CPFE, neutrophil elastase and matrix metalloproteinases, enzymes physiologically involved in the destruction of bacteria by degrading the cell wall and in remodeling the cell–matrix by dissolving collagen, exceed the capacity of natural α1-antitrypsin inhibitors and lead to the destruction of alveolar septa rich in elastic fibers, resulting in emphysema [13]. The persistence of oxidative stress and inflammation causes an additional release of profibrotic cytokines such as TGF-β1, TNF-α and PDGF, which activate fibroblasts and cause their differentiation into myofibroblasts, with excessive collagen accumulation and interstitial remodeling, resulting in pulmonary fibrosis [6,17]. The overlap of these proteolytic and profibrotic processes contributes to the development of a mixed emphysematous-fibrosing pattern characteristic of CPFE [2].

Chronic exposure to smoking and pollutants leads to a marked imbalance between protease and antiprotease, especially if there is already an alpha1-antitrypsin deficiency found in COPD or through the activation of elastases, mechanisms that lead to extracellular matrix degradation, septal loss and subsequent hyperinflation [13]. At the same time, oxidative stress caused by cigarette smoke generates endothelial dysfunction and apoptosis, with negative changes on the alveolar–capillary membrane [18].

In pulmonary fibrosis, through the TGF-β factor as the main fibrotic agent, there is a reduction in lung elasticity and additional thickening of the alveolar–capillary membrane, which causes progressive ventilatory restriction [17].

Pulmonary vascular remodeling, represented by smooth muscle cell proliferation, contributes to the development of PH, a major negative prognostic factor among patients who develop CPFE [4].

The genetic component plays an important role in increasing the susceptibility to the development of this syndrome. Listed genetic polymorphisms such as MMP-9, TGF-β1 or AGER and mutations in genes involved in the maintenance of telomere length (TERT, TERC) can favor mucus hyperproduction, alteration of mucociliary clearance and the occurrence of repeated alveolar microinjuries with defective repair of the alveolar epithelium, favoring the transition from acute inflammation to fibrosis [2,9,10]. Telomere shortening reflects an aging of lung cells and contributes to a decrease in the regenerative capacity of type II pneumocytes, promoting an irreversible fibrotic phenotype [9,10].

**Figure 1:**
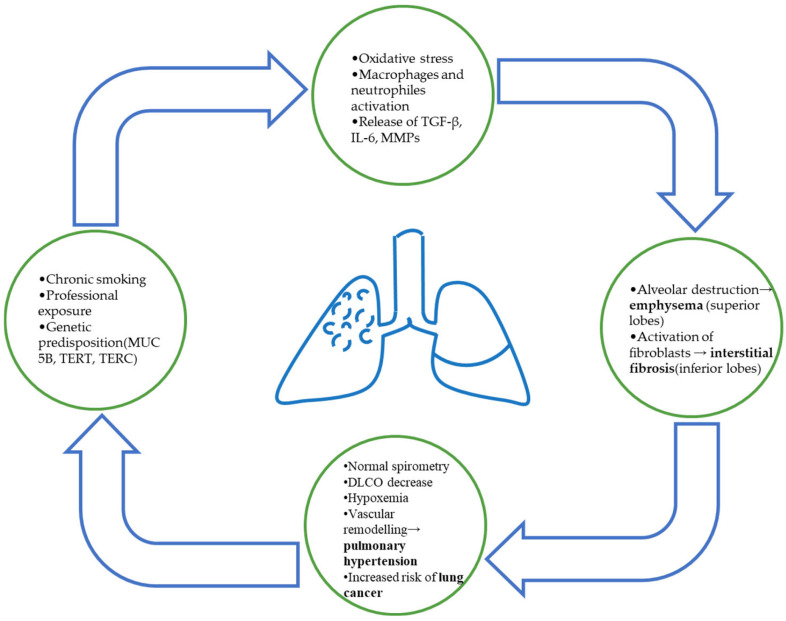
Schematic representation of CPFE pathophysiology. Chronic smoking and environmental exposure lead to oxidative stress and inflammatory activation, resulting in simultaneous alveolar destruction (emphysema) and fibrotic remodeling (interstitial fibrosis). The coexistence of both processes contributes to impaired gas exchange, PH, and increased risk of lung cancer.

The pathophysiological process linking emphysema and fibrosis remains incompletely understood. Controversies exist regarding the role of smoking compared with genetic predisposition and cellular aging in triggering the fibrotic process. Furthermore, most data come from studies on small numbers of subjects or experimental studies, limiting the validity of the results and indicating the need for further research. Figure 1 shows a schematic representation of the main mechanism involved in the development of CPFE.

### 3.5. Radiological Findings

Chest radiography in CPFE may reveal an interstitial or reticulonodular pattern at the lung bases or at the subpleural level, but also hypertransparent at the apex, accompanied by thinning of the pulmonary vessels and a reduction in their number [2,15]. This topography seems to reflect chronic exposure to noxious substances, as well as the increased susceptibility of different lung areas to inflammatory mechanisms [15].

In the upper lobes, excessive destruction of the alveolar septa through the activation of proteases and α1-antitrypsin deficiency favors the development of emphysema [13]. In the lower lobes, repeated alveolar injuries and the release of profibrotic cytokines lead to the activation and differentiation of fibroblasts into myofibroblasts, a central mechanism in fibrogenesis [6,17].

Radiological changes observed on chest radiography are not diagnostic, which is why HRCT evaluation is necessary for confirmation [2,15]. Also, early fibrosis or small centrilobular emphysema may be missed on chest radiography, which is why it is mainly used as a screening test [15]. The accuracy of HRCT investigation contributes not only to diagnosis, but also to the early detection of potential complications (PH, bronchopulmonary carcinoma or superimposed infections) [2,3,4].

There are certain radiological criteria that underlie the definition of CPFE, including: the presence of emphysema on HRCT, defined as an area of low attenuation, delimited by the adjacent normal lung and with very thin (<1 mm) or even absent walls, as well as the presence of multiple bullae (>1 cm), predominantly superior [15]. The presence of architectural distortion, honeycombing, bronchiectasis or traction bronchioles, as well as reticular infiltrates, predominantly basal or subpleural, are also part of the radiological definition of CPFE, which also requires the presence of interstitial lung disease [2,3,4,15,16].

In a cohort analyzed by Wong et al., approximately 65–70% of patients with CPFE had large bullae at the lung apex [15]. Also, patients with more than 20% of the parenchyma affected by fibrosis had a higher mortality and an increased risk of PH [2,14]. Imaging can capture complications developed by the patient, being reflected by dilation of the pulmonary arteries, the appearance of vascular plexuses, solid or cavitary nodules, or changes compatible with superimposed infections [1,12,16]. In 20–25% of cases, fibrosis is not evident on a standard chest radiography, which highlights the need for screening with HRCT for diagnostic accuracy and monitoring [14,16].

New radiomics and artificial intelligence techniques allow for the automatic characterization of fibrotic changes and emphysema, thus helping to stratify patients into clinical subtypes: emphysema-dominant, fibrosis-dominant and vascular-dominant. Models have demonstrated up to 82% accuracy in determining CPFE phenotypes, suggesting a role in personalized case management [11,13]. HRCT provides valuable diagnostic information and can detect and characterize early potential complications and guide personalized therapeutic strategies [1,2,14].

### 3.6. Symptomatology of CPFE

CPFE is characterized by a distinctive clinical profile that integrates epidemiological, symptomatic, and systemic features, which together shape prognosis. The strong association with tobacco exposure appears to unify CPFE with both emphysema and interstitial lung disease, although occasional cases occur in nonsmokers [1,19]. Dyspnea is the predominant symptom, being reported in more than 95% of patients [15,20]. It typically progresses insidiously, with exertional breathlessness emerging years before diagnosis.

Dyspnea severity is disproportionate to spirometric impairment and often advances despite relatively preserved lung volumes, with up to 80% of patients reaching advanced stages of exertional limitation during follow-up [2,21,22]. Chronic cough is another often observed feature, present in 30–70% of cases [1,23]. While dry in some patients, cough may be persistent and associated with recurrent exacerbations, reflecting the dual fibrotic-emphysematous substrate [15,20].

Effort tolerance is typically reduced, with patients experiencing early exercise-induced desaturation. In observational cohorts, more than 60% desaturated below 90% SpO_2_ on standardized exertional testing, correlating with pulmonary vascular involvement [2,19]. Auscultatory findings are also distinctive: bilateral basal velcro crackles are nearly universal, reported in 87–100% of cases [2,23], while wheezing is less frequent and generally reflects airway comorbidities [19,24].

Systemic and extrapulmonary manifestations further define the clinical phenotype. Digital clubbing is observed in 40–67% of patients [1,23], while cyanosis—exertional or resting—appears in advanced disease and correlates with the development of PH [2,21]. Lung cancer complicates up to 46% of CPFE cases [2,15,23], with squamous cell carcinoma being the most frequent histological type. The prognosis is poor, as survival following lung cancer diagnosis in CPFE rarely exceeds 12 months [15,23]. Acute exacerbations, although less common than in idiopathic pulmonary fibrosis (IPF), are of a great impact when they occur, carrying a hospital mortality rate exceeding 50% [19,22].

Beyond pulmonary complications, multisystemic comorbidities are highly prevalent and do shape the clinical profile of each patient. Cardiovascular disease is present in nearly half of CPFE patients [20], consistent with the synergistic effects of smoking, hypoxemia, and PH. Metabolic disorders such as diabetes and systemic hypertension also appear more frequently in CPFE cohorts than in COPD or IPF alone, reflecting shared risk factors and systemic inflammation [15]. Collectively, these comorbidities exacerbate exercise intolerance, drive clinical decline, and contribute to the heterogeneity of patient trajectories.

The natural history of CPFE is one of relentless decline. Progressive dyspnea, increasing dependence on supplemental oxygen, and recurrent hospitalizations for exacerbations mark the clinical course. Median overall survival is highly variable but consistently shorter in patients with complications such as PH or lung cancer.

Observational studies report 5-year survival rates ranging from 35% to 55%, values lower than those seen in emphysema but comparable or slightly worse than in isolated pulmonary fibrosis [1,2,21,25].

### 3.7. Pulmonary Function Tests (PFTs)

PFTs play a key role in characterizing CPFE, offering a functional correlation to its distinctive radiological and pathological features. One central finding is the near-normal static lung volumes, despite extensive parenchymal abnormalities. Multiple cohorts consistently report preserved or only mildly reduced FVC (80–97% predicted) and TLC (90–102% predicted), reflecting the *counterbalancing* effects of fibrotic restriction and emphysematous hyperinflation [2,19,20]. In contrast, isolated idiopathic pulmonary fibrosis (IPF) typically presents with a marked reduction in FVC (60–70% predicted) and TLC (65–75% predicted) [19,20], while COPD is characterized by hyperinflation with increased TLC and residual volume [15]. In CPFE, the FEV1/FVC ratio is often normal or only mildly decreased (70–75%) [2,19], diverging from the classic obstructive signature of COPD, where values fall below 70% [15].

The main physiological abnormality in CPFE lies in diffusing capacity. Across several studies, DLCO is consistently and severely reduced, averaging 35–45% predicted [2,19,20,21], which is lower than in COPD (50–60% predicted) [15] and at least comparable, if not more impaired, than in IPF (40–50% predicted) [19,20]. In a cohort study, 87% of patients with CPFE had a DLCO < 50% predicted, compared to 68% of IPF and 47% of COPD patients [2]. This disproportionate reduction in gas transfer is attributed to the combined destruction of alveolar–capillary surface by emphysema and interstitial thickening by fibrosis, hence the frequent occurrence of severe resting and exertional hypoxemia.

Exercise testing further emphasizes functional compromise. In a large cohort, mean 6-min walk distance in CPFE was 298–320 m, significantly shorter than in COPD (380–420 m) and IPF (340–360 m) [20,21,23]. Moreover, desaturation during 6MWT is profound, with a mean reduction in SpO_2_ of 8–10%, and over 70% of CPFE patients desaturating below 88%, compared with 40–50% in IPF and 30–40% in COPD [2,21,23]. Cardiopulmonary exercise testing reveals severely reduced peak VO_2_ (10–13 mL/kg/min, 45–55% predicted) and elevated VE/VCO_2_ slope (38–42), both worse than in COPD and IPF [21,23]. These exercise limitations do correlate with pulmonary vascular involvement, which is more prevalent in CPFE than in either COPD or IPF alone [19,23].

Regarding the prognostic, functional indices in CPFE provide independent survival signals. Severely reduced DLCO (<30% predicted) and significant 6MWT desaturation (>10% reduction in SpO_2_) are associated with markedly worse outcomes, described by hazard ratios for mortality ranging from 1.8 to 2.3 in multivariable models [2,19,21].

The preserved FVC in CPFE may therefore mask disease severity, making DLCO and exercise desaturation more reliable prognostic indices than spirometry alone [2,19,23]. Compared with COPD and IPF, patients with CPFE demonstrate a more rapid functional decline in gas transfer and exercise capacity, while spirometric changes progress more slowly [2,20].

CPFE is defined by a paradoxical PFT pattern of preserved volumes, near-normal spirometry, and disproportionately reduced DLCO, combined with profound exercise-induced gas exchange impairment. This functional profile distinguishes it both from COPD, where airflow obstruction and hyperinflation dominate, and from IPF, where restrictive volumes are the primary alteration. However, in CPFE, the combined insult results in greater physiological impairment, particularly regarding the gas transfer and exercise limitation, underscoring the fall in the quality of life and also the poor prognosis.

### 3.8. Complications—Beyond Fibrosis and Emphysema

The clinical burden of CPFE extends far beyond the dual impairment of gas exchange and structural damage, encompassing a spectrum of severe complications that critically influence outcomes. Among these, PH, acute exacerbations, and malignancy dominate can shape the unfavorable prognosis characteristic of CPFE.

PH is one key complication in CPFE and is significantly more frequent than in isolated idiopathic IPF or COPD. Studies consistently report PH prevalence rates between 47% and 90% in CPFE patients [1,2,15,19], compared to approximately 31–46% in IPF and 23–40% in COPD [20,21]. Hemodynamically, mean pulmonary arterial pressures in CPFE often exceed 35 mmHg, with 47% of patients reaching this threshold in one case series [2]. PH in CPFE arises from the additive effects of fibrotic vascular distortion, emphysematous loss of vascular bed, and hypoxic vasoconstriction [1,2]. The presence of PH confers a strong prognostic penalty, with median survival falling to 25 months in CPFE patients with confirmed PH compared to 54 months in those without [2]. Notably, combined data indicate a two-fold higher risk of death when PH is present, reinforcing its role as the most important prognostic determinant in CPFE [2,19,23].

Acute exacerbations (AEs) represent another life-threatening complication, with a frequency comparable to IPF but potentially higher severity due to the coexistent emphysema [15,20]. Reported AE incidence ranges between 20% and 28% over follow-up periods of 1–3 years [20,23]. These events are thought to reflect diffuse alveolar damage superimposed on fibrotic and emphysematous architecture, with impaired mechanical reserve compounding hypoxemia. The clinical consequences are visible: in-hospital mortality after AEs reaches 60% in CPFE, compared to approximately 50% in IPF [23]. Additionally, AEs in CPFE frequently follow infectious triggers, possibly reflecting the vulnerability of damaged parenchyma to secondary insults [20].

The risk of lung cancer is particularly high in CPFE, exceeding those reported in both IPF and COPD. Lung cancer prevalence in CPFE cohorts ranges from 22% to 47%, compared to 7–20% in IPF and 12–16% in COPD [1,19,24]. Squamous cell carcinoma is the most common histological type, representing nearly 50–52% of cancers diagnosed in CPFE patients, followed by adenocarcinoma at 34–35% and small cell lung carcinoma in 8–9% [19,24]. The increased incidence has been attributed to a synergistic interplay between smoking-related mutagenic injury, emphysematous microenvironment, and fibrotic epithelial remodeling [1,19]. Prognostically, CPFE-associated lung cancer carries worse outcomes, with five-year survival rates below 20% even when adjusted for stage [24].

Other complications include recurrent infections, chronic respiratory failure, and cardiovascular disease. Infections are facilitated by impaired mucociliary clearance in emphysematous regions combined with fibrotic distortion [1,20]. Chronic respiratory failure is almost inevitable as disease progresses, with reported long-term oxygen therapy requirements in up to 55–60% of patients [2,15]. Cardiovascular morbidity is elevated due to shared smoking-related risk factors and hypoxemia-driven vascular stress, and in general regards the chronic cor pulmonale and ischemic heart disease [19,21]. Together, these complications add a cumulative burden that extends beyond the primary fibrotic-emphysematous damage, contributing to the distinctive lethality of CPFE.

Data on CPFE complications are heterogeneous, with major differences between studies regarding the prevalence of PH and lung cancer. These discrepancies reflect the absence of universally accepted evaluation and diagnostic criteria. Prospective studies remain necessary to validate major prognostic factors.

### 3.9. Differential Diagnosis and Overlapping Entities in CPFE

The recognition of CPFE has substantially improved in the last decade, yet distinguishing it from other chronic lung diseases still is a clinical challenge. Misclassification is not uncommon, as many of its functional and radiological features overlap with those of COPD, IPF, and other diffuse parenchymal lung diseases. Up to 25–30% of patients initially suspected of having COPD or IPF may actually meet the criteria for CPFE when both emphysematous and fibrotic patterns are systematically assessed through HRCT and comprehensive pulmonary function testing [21,22].

One diagnostic issue arises in distinguishing CPFE from COPD. Both entities share a smoking-related background, but the distribution and physiological impact of emphysema differ. Patients with CPFE often show preserved or near-preserved FEV1/FVC ratios despite advanced parenchymal damage, whereas COPD typically demonstrates a marked reduction in this parameter [21,22]. In a pooled analysis, 65–80% of CPFE patients had FEV1/FVC ratios within the normal range, compared with less than 20% of COPD patients, a discrepancy that underscores the risk of underdiagnosing the fibrotic component if spirometry alone is used [21,25]. Moreover, DLCO provides a reliable discriminator: severe reduction in DLCO is observed in more than 90% of CPFE cases, often disproportionate to spirometric impairment, while COPD usually presents with milder declines [22]. Misinterpretation of these patterns can delay recognition of CPFE, with prognostic implications given its higher frequency of PH and malignancy [21].

Differentiation from IPF presents a different set of challenges. Radiologically, both entities share basal reticular opacities and honeycombing, yet the upper-lobe-predominant emphysema in CPFE alters both the functional trajectory and clinical course. In a cohort-based comparison, patients with CPFE exhibited significantly higher TLC and RV values than those with isolated IPF, despite similar degrees of fibrosis, suggesting that emphysematous destruction counterbalances the restrictive effect of fibrosis [22]. This apparent *normalization* of lung volumes may mask the severity of fibrosis, particularly when HRCT findings are subtle, contributing to under-recognition in up to 15% of cases [21]. Clinically, both diseases present with exertional dyspnea, but oxygen desaturation during exercise is generally more profound in CPFE, reflecting the combined gas-exchange burden [2]. Failure to recognize this dual pathology may lead to inappropriate application of IPF-only treatment strategies.

Additional overlap occurs with other ILDs, particularly chronic hypersensitivity pneumonitis and connective tissue disease-associated ILDs, in which mixed emphysematous and fibrotic patterns can also arise [22]. The distinction is clinically relevant, as management differs, but in imaging studies, emphysema tends to be less extensive and not as sharply upper-lobe-predominant as in CPFE. Furthermore, a history of significant smoking exposure, reported in over 90% of CPFE patients across studies, strongly favors this diagnosis over other ILDs [21,22].

**Figure 2:**
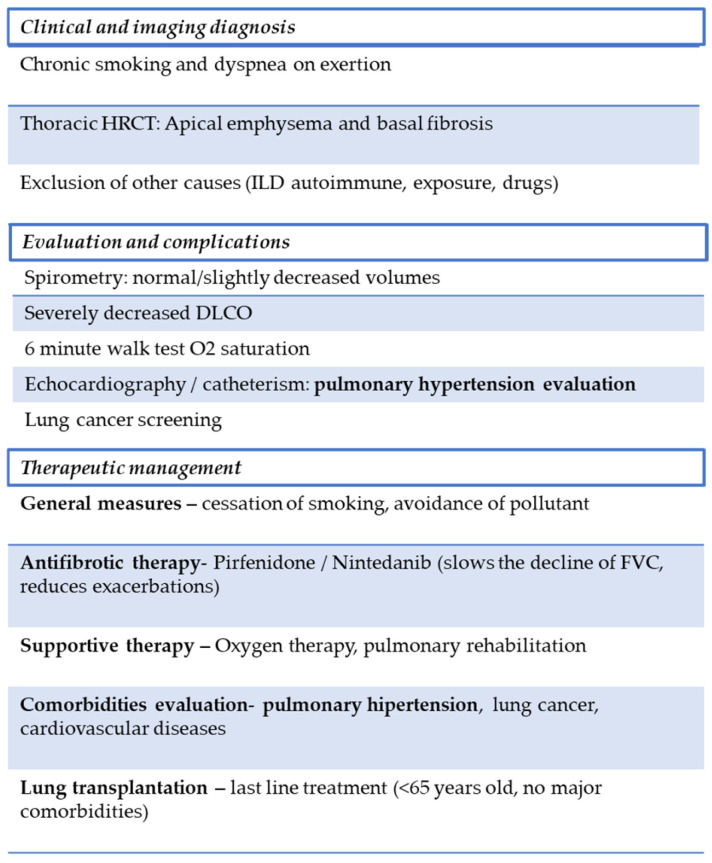
Proposed diagnostic and management flowchart for CPFE. The diagnostic process integrates clinical history, HRCT findings, and functional assessment. Management focuses on smoking cessation, antifibrotic therapy when appropriate, and treatment of major complications such as PH and lung cancer. A multidisciplinary approach is essential to improve outcomes.

The risk of misdiagnosis is amplified in clinical practice settings that rely heavily on spirometry without integrating HRCT and DLCO measurements. Up to one-third of patients classified as COPD based on symptoms and spirometry alone were later reclassified as CPFE once HRCT revealed concurrent fibrosis [2,21]. Conversely, patients labeled with IPF may have their emphysematous burden overlooked, particularly when the fibrotic component dominates symptomatology. Establishing a clear framework for differential diagnosis thus requires a multimodal approach. HRCT remains the cornerstone for imagistic confirmation, but functional parameters—specifically preserved spirometry with disproportionately reduced DLCO—provide a strong physiological clue for CPFE [2,22]. As shown in Figure 2, a schematic representation of the diagnostic and management approach in combined pulmonary fibrosis and emphysema is presented.

### 3.10. Current and Emerging Strategies in CPFE Management

The therapeutic management of CPFE remains a challenge, largely due to the absence of a standardized treatment framework, which often leads to delayed interventions and inconsistent patient outcomes [1,2,20]. The absence of consensus guidelines contributes to variability in clinical practice, with therapeutic decisions frequently extrapolated from COPD or IPF strategies, despite CPFE representing a distinct clinical entity [2,19]. This uncertainty has tangible prognostic consequences, as patients with CPFE exhibit higher rates of respiratory failure, PH, and lung cancer than those with isolated COPD or IPF [15,21,23].

Smoking cessation remains a cornerstone of management, given the strong etiological link between tobacco smoke exposure and CPFE. More than 90% of affected patients report heavy smoking histories, and cessation has been associated with attenuation of emphysema progression and improved functional stability [20]. However, adherence is inconsistent, and even after cessation, fibrosis may progress, underscoring the need for adjunctive therapies [20,22].

Oxygen therapy is frequently required, as over 70% of CPFE patients develop hypoxemia during disease progression [20]. The need for oxygen supplementation typically increases over time, with flows escalating from nocturnal or exertional use to continuous daytime administration [21]. This rise in oxygen demand is a marker of worsening fibrosis and pulmonary vascular involvement, and long-term oxygen therapy has been associated with reduced exacerbation frequency and improved exercise tolerance. Nevertheless, survival benefits remain less pronounced compared with antifibrotic therapy, reflecting the supportive rather than disease-modifying role of oxygen [2].

Antifibrotic treatment represents one of the few interventions with disease-modifying potential. Evidence derived from patients with IPF and emphysema overlap indicates that agents such as pirfenidone and nintedanib slow the decline in FVC by approximately 50% compared to placebo [2]. In CPFE, similar trends have been observed, with stabilization of FVC decline and delayed acute exacerbations [2,21]. Nonetheless, the impact of antifibrotics on survival remains modest, with mortality still heavily influenced by complications such as PH and lung cancer [15,23,25]. Even so, antifibrotics currently represent the only pharmacological intervention shown to significantly alter disease trajectory, thereby exerting the largest impact on long-term prognosis [2].

Emerging antifibrotic strategies are increasingly directed toward molecular targets implicated in interstitial remodeling. This may present a potential translational relevance for CPFE management. Among these, phosphodiesterase-4B inhibition has been recently explored as a therapeutic pathway. Nerandomilast, an oral selective PDE4B inhibitor, demonstrated attenuation of FVC decline over 52 weeks when administered either as monotherapy or in combination with approved antifibrotic therapy (−114.7 mL in medicated groups versus, −183.5 mL in placebo group). Therefore, a possibility arises of additive benefit in fibrosing phenotypes characterized by inflammatory activation [26]. The study also emphasized tolerability aspects. In this regard, the most frequent adverse event in the nerandomilast groups was diarrhea, reported in 31.1–41.3% of the medicated group, as compared with 16.0% in the placebo group. This should be considered, as CPFE patients present multiple comorbidities that can be aggravated by dehydration and dyselectrolytemia, such as cardiac failure [19,21]. Parallel investigation of the autotaxin-lysophosphatidic acid axis has given novel compounds with antifibrotic potential. The ISABELA-1 and ISABELA-2 phase 3 trials evaluated ziritaxestat, an autotaxin inhibitor, in idiopathic pulmonary fibrosis, thus demonstrating the feasibility of large-scale testing of pathway-specific agents. However, the findings argue for cautious interpretation of early-phase signals and for designing CPFE-specific trials rather than extrapolating from IPF alone [27]. Also, earlier work identified selective autotaxin inhibitors through DNA-encoded chemistry approaches, offering a model for precision drug discovery applicable to mixed fibrotic-emphysematous diseases.

Evidence from real-world cohorts further supports the role of antifibrotic therapy across progressive fibrosing interstitial lung diseases. Observational data have shown stabilization of lung function and slower progression irrespective of the initiating cause, providing reason for extending therapeutic evaluation to CPFE populations [28], although specific cohorts should be studied. Another emerging aspect of antifibrotic therapies are the inhaled delivery platforms, such as nanoparticles, microparticles and targeted carriers. The platforms are to be used as a route to deliver antifibrotic agents directly to the lung, potentially improving local efficacy and reducing systemic toxicity. Findings show inhalation as a promising strategy for agents with systemic tolerability issues (relevant given gastrointestinal adverse events observed with some oral agents), and for combining antifibrotic molecules with targeted carriers to reach fibrotic foci. Moreover, the treatment adherence in chronic multimorbidity conditions, such as CPFE (the major demographic category being older male smokers [16]), represents a challenge that inhaled delivery platforms may address in a rather efficient way [29].

Management of complications in CPFE requires personalized interventions to alleviate their prognostic impact. PH may be stabilized with vasodilators and oxygen therapy, improving exercise tolerance though long-term survival benefit remains modest [2,21]. PH in CPFE can be palliated and in some cases partially stabilised with targeted pulmonary vasodilators and long-term supplemental oxygen, leading to measurable improvements in exercise tolerance. However, consistent long-term survival benefit remains modest. Inhaled treprostinil produced a statistically significant improvement in 6-min-walk distance in the phase-3 INCREASE trial, showing a placebo-corrected median improvement of approximately 21 m by Hodges-Lehmann estimate and a mixed-model mean of 31 m with *p* < 0.004. This, combined with reductions in NT-pro-BNP and fewer clinical-worsening events led to FDA approval for treatment of PH-ILD [30].

Smaller studies and registry data suggest phosphodiesterase-5 inhibitors (PDE5i, such as sildenafil) may preserve exercise capacity and quality of life in patients with IPF and documented right ventricular dysfunction, but results have been heterogeneous and benefit appears primarily symptomatic rather than convincingly mortality-changing. Clinical evidence on sildenafil in fibrotic lung diseases and PH remains mixed, with some promising signals but no definitive benefit in key outcomes. One of the earliest open-label ILD-PH experiences enrolled 15 patients (mean age 55 ± 15 years; mean FVC 52.6 ± 15.4%) with ILD and documented PH (mean RV systolic pressure 73.8 ± 17.8 mmHg). After six months of sildenafil therapy, BNP levels fell (*p* = 0.03) and six-minute walk distance increased (*p* < 0.05), but no change in right ventricle systolic pressure was recorded [31]. The only large randomised controlled trial in advanced IPF assessed 180 patients over 12 weeks: 9/89 (10%) in sildenafil vs. 6/91 (7%) in placebo achieved a ≥ 20% increase in six-minute walk distance (*p* = 0.39). While small improvements in oxygenation, diffusion capacity and dyspnea were observed favouring sildenafil, the primary endpoint was not met [32]. As for the survival rate in ILD-PH more broadly, a cohort of 128 patients (50 treated with PDE5i) reported median survival 2.18 years (95% CI: 1.43–3.04) in treated versus 0.94 years (0.69–1.51) in untreated [33].

Lung cancer, affecting up to 46% of the patients, is approached with surgery, chemotherapy or immunotherapy, yet outcomes remain poor with median survival under a year [15,23]. Acute exacerbations demand high-dose corticosteroids and ventilatory support, but hospital mortality still exceeds 50% [19,22]. These strategies highlight the limits yet necessity of aggressive, complication-specific treatment.

Current literature on CPFE management is limited and lacks randomized clinical trials specifically dedicated to this clinical entity. Most therapeutic recommendations are derived from the treatment of patients with idiopathic pulmonary fibrosis. This approach creates uncertainty regarding the actual effectiveness of antifibrotic agents and vasodilator therapy in CPFE, highlighting the need for dedicated studies to evaluate their impact on survival.

Pulmonary transplantation represents the ultimate treatment for eligible patients, particularly those under 65 years of age without severe comorbidities [24,34]. Post-transplant survival in CPFE parallels that of IPF, with 5-year survival rates between 50% and 55% [24]. However, only a minority of patients qualify due to advanced age, smoking-related cardiovascular disease, or malignancy [34]. As such, transplantation, while offering the most definitive survival benefit, is restricted to a select subset of patients.

### 3.11. Emerging Tools and Future Directions

The future management of CPFE is shifting from descriptive recognition toward precision-based interventions, integrating clinical, imaging, physiological, and molecular domains. Serum biomarkers reflecting epithelial injury, extracellular matrix remodeling, and systemic inflammation have been investigated for distinguishing CPFE from IPF or emphysema, though none have achieved clinical validation. Circulating matrix metalloproteinases were higher in CPFE compared with fibrosis-predominant ILD, indicating parenchymal turnover distinct from IPF [2,20]. KL-6 and SP-D levels were frequently elevated, with KL-6 exceeding 1000 U/mL in >40% of patients, suggesting potential subgroup differentiation [20,21]. Endothelin-1 correlated with pulmonary vascular involvement, a complication affecting 47–55% of cases [2,23]. Elevated brain natriuretic peptide and troponins were reported in up to 42% of CPFE patients with PH, supporting integrated cardiopulmonary biomarker panels [19,23].

On a molecular level, pathways involving TGF-β signaling, endothelial injury, and extracellular matrix remodeling have been repeatedly described in CPFE, overlapping but not identical to those in IPF or COPD [21]. Potential therapeutic targeting of these mechanisms, including experimental inhibitors of integrins, oxidative stress mediators, antifibrotic agents and modulators of vascular remodeling, may represent future directions for disease-specific interventions [20,21].

CPET is gaining relevance as an integrative tool in CPFE evaluation. In a comparative study, CPFE patients demonstrated peak VO_2_ reductions of nearly 35% compared with predicted values, more severe than emphysema-predominant COPD but distinct from IPF alone. CPET-derived ventilatory equivalents (VE/VCO_2_ slope) were elevated in more than 60% of subjects, indicating exercise-induced circulatory limitation [15]. Coupling CPET with echocardiography or invasive hemodynamics may identify patients at higher risk of vascular complications, particularly PH, which is documented in 47–55% of advanced CPFE cohorts [21,22].

Serum matrix metalloproteinase-7 (MMP-7) has emerged as a reproducible indicator of fibrotic progression in interstitial lung disease, with concentrations above 3.53 ng/mL associated with increased risk of progression and an adjusted odds ratio of approximately 1.26 for fibrotic worsening [35]. More MMP/TIMP (matrix metalloproteinase/tissue inhibitors of metalloproteinases) have demonstrated diagnostic utility in connective tissue disease-related ILD, particularly MMP-2, -7, -9, -10, -12 and TIMP-1, which, when analyzed in multi-marker panels, outperform single parameters in differentiating early fibrotic changes [36]. This suggests that similar combinatorial panels could improve early stratification in CPFE, capturing both emphysematous and fibrotic remodeling dynamics.

Circulating microRNAs, particularly miRNA-486, show diagnostic potential for right ventricular remodeling, achieving an AUC of approximately 0.86 in distinguishing maladaptive from adaptive responses [37]. Their integration into CPFE research may improve early detection of PH and right-heart dysfunction, the major determinants of cardiac prognostic. Complementary reviews reiterate the need for such multi-marker panels, emphasizing that combining fibrotic (MMPs, collagen fragments), epithelial (KL-6, SP-D), and cardiopulmonary (BNP, miRNAs) markers with imaging and physiological indices may create more robust predictive frameworks [38].

Artificial intelligence represents another avenue for refinement. Automated CT quantification already improves emphysema and fibrosis scoring reproducibility. In CPFE, hybrid radiomic models integrating texture features with clinical data achieved an accuracy of 0.82 in distinguishing emphysema-dominant versus fibrosis-dominant phenotypes [2,24]. These approaches may eventually support phenotype-driven clinical trials, overcoming the current heterogeneity in treatment response.

The recognition of sub-phenotypes—emphysema-dominant, fibrosis-dominant, and vascular—would also be reliable. Emphysema-dominant patients, representing nearly one-third of cohorts, demonstrate preserved lung mechanics with slower fibrotic progression, but severe gas exchange abnormalities, while fibrosis-dominant disease, found in 40–45% of cases, aligns closer to IPF biology [2,21]. Vascular phenotypes, marked by PH in up to half of patients, show the highest biomarker burden and exercise impairment [22,23]. These patterns indicate the need for distinct therapeutic pathways.

Lung transplantation remains a viable therapeutic endpoint. Although underreported, CPFE patients account for 5–7% of all ILD transplants in recent registries, with survival outcomes similar to IPF when matched for comorbidities (1-year survival exceeding 70%) [25]. Nevertheless, the coexistence of emphysema raises perioperative challenges, reinforcing the need for phenotype-sensitive selection criteria.

There is also a growing call for more specific diagnostic scores. Current criteria, based largely on imaging and clinical context, may be refined by integrating biomarker panels, quantitative CT, and CPET-derived indices into multiparametric frameworks, with standardized thresholds. The inherently complex nature of CPFE requires multidisciplinary management. Pulmonologists, cardiologists, oncologists, and vascular medicine specialists must converge in decision-making, as patients frequently present overlapping features of parenchymal destruction, fibrotic remodeling, PH and malignancy [2,19]. This integrated perspective may ultimately provide the structure necessary for a unified therapeutic framework in CPFE.

## 4. Conclusions

CPFE is a distinct clinical entity characterized by an atypical functional, imaging, and pathophysiological profile that cannot be fully explained by COPD or fibrosis alone. It is often underestimated and underdiagnosed due to relatively preserved spirometric values and disproportionately reduced DLCO. While male smokers are predominantly affected, CPFE should not be excluded in patients with connective tissue diseases or those with occupational exposure to harmful agents. Genetic evaluation, when other causes have been ruled out, can provide valuable insights into both susceptibility and prognosis. HRCT remains essential for diagnosis, alongside functional changes that offer independent prognostic value. In CPFE, disproportionate drops in oxygen saturation during exertion and smoking-related desaturation are stable and clinically relevant markers of mortality, often providing more informative prognostic value than spirometry.

With the rising number of smokers, CPFE represents a clinically significant condition that increases the risk of developing interstitial lung diseases (ILDs). Early recognition of affected individuals is critical, as timely diagnosis can substantially improve prognosis and guide future treatment strategies. Smokers, particularly men, are the most affected group, and monitoring disproportionate functional markers such as DLCO and exercise-induced desaturation can offer prognostic information that is often more informative than spirometric values. Additionally, there are still considerable gaps in the literature regarding the molecular mechanisms, CPFE subphenotypes, and the role of genetic testing. This presents opportunities for translational research, as well as the integration of advanced technologies—such as biomarkers, CT imaging, and cardiopulmonary exercise testing (CPET)—to optimize treatment and improve both survival and quality of life.

The disease significantly shortens life expectancy through the development of PH, progressive respiratory failure, and, ultimately, lung cancer. Ongoing, careful monitoring, along with the combined use of imaging, functional, and genetic assessments, remains crucial for managing CPFE. However, clinical management remains challenging. Lung transplantation is still the only definitive treatment for affected patients. Future research is focusing on precision medicine, integrating biomarkers, automated CT quantification, CPET, and radiomic models to identify subphenotypes and guide treatment. Such approaches hold the potential to improve both survival and quality of life, especially in the context of a growing smoking population and the increasing clinical relevance of CPFE.

## Data Availability

The original contributions presented in this study are included in the article. Further inquiries can be directed to the corresponding author.
